# Unraveling the Adipose Tissue Proteome of Transition Cows through Severe Negative Energy Balance

**DOI:** 10.3390/ani9121013

**Published:** 2019-11-21

**Authors:** Cristian Piras, Valeria Maria Morittu, Anna Antonella Spina, Alessio Soggiu, Viviana Greco, Christelle Ramé, Eric Briant, Namya Mellouk, Bruno Tilocca, Luigi Bonizzi, Paola Roncada, Joëlle Dupont

**Affiliations:** 1 Department of Chemistry, University of Reading, Reading RG66AH, UK; c.piras@reading.ac.uk; 2 Dipartimento di Medicina Veterinaria, Università degli Studi di Milano, 20133 Milano, Italy; alessio.soggiu@unimi.it; 3 Department of Health Sciences, University Magna Graæcia, 88100 Catanzaro, Italy; morittu@unicz.it (V.M.M.); anto.spina90@gmail.com (A.A.S.); tilocca@unicz.it (B.T.); 4 Istituto di Biochimica e Biochimica Clinica, Università Cattolica del Sacro Cuore, 00168 Roma, Italy; viviana.greco@unicatt.it; 5 Fondazione Policlinico Universitario A. Gemelli IRCCS, 00168 Roma, Italy; 6 Department of Animal Physiology and Livestock Systems, French National Institute for Agricultural Research—INRA, F-37380 Nouzilly, France; Christelle.rame@inra.fr (C.R.); eric.briant@inra.fr (E.B.); mellouk.namya@nih.gov (N.M.); 7 Dipartimento di Scienze Biomediche, Chirurgiche ed Odontoiatriche, Università degli Studi di Milano, 20133 Milano, Italy; luigi.bonizzi@unimi.it

**Keywords:** energy requirements, peripartum, fat mobilization, proteomics

## Abstract

**Simple Summary:**

This work described the analysis of differential protein expression of subcutaneous adipose tissue of cows that went under negative energy balance during peripartum. In particular, to the best of our knowledge, it represented an original proteomics study that was able to discriminate cows in negative energy balance up to one month before calving. We believed that our findings would open new perspectives to improve animal welfare during peripartum. To know in advance, the metabolic status of cows would permit to correct the status with appropriate measures, like diet or management.

**Abstract:**

Fat mobilization in high-yielding dairy cows during early lactation occurs to overcome negative energy balance (NEB), caused by insufficient feed intake and the concomitant increased nutritional requirements. For this reason, adipose tissue represents an essential organ for healthy and performant lactation. However, only a few data are known about adipose tissue proteome and its metabolic status during peripartum. The aim of this study was to analyze the differential proteomics profiles of subcutaneous adipose tissue belonging to cows with different NEB scores (low NEB and severe NEB). Both groups were analyzed at three different time points (one month before calving, one and sixteen weeks after calving) that were related to different levels and rates of adipose tissue mobilization. The dataset highlighted the differential expression of the same four key proteins (annexin A2, actin-related protein 10, glyceraldehyde-3-phosphate dehydrogenase, and fatty acid-binding protein) involved in lipid metabolism during all time points and of other 22 proteins typical of the other comparisons among remaining time points. The obtained dataset suggested that the individual variability in adipose tissue metabolism/mobilization/energy availability could be linked to the different outcomes in levels of energy balance and related physical complications among dairy cows during peripartum.

## 1. Introduction

After parturition, the energy intake of high-yielding dairy cows is often not sufficient to meet the elevated energy requirements for milk production. Body energy reserves, in particular, adipose tissue, are mobilized to meet this energy deficit, and this state is often referred to as negative energy balance (NEB). With the mobilization of 50 to 60 kg of adipose tissue during early lactation, lipolysis is thus the essential metabolic process to support energy supply for all physiological functions [1]. It is well known that prolonged NEB can reduce overall fitness. Declining reproductive fitness is one of the main reasons leading to culling decisions in cattle [2]. Indeed, various measurements of reproduction, including days to first ovulation and days open, have been negatively associated with severe or prolonged NEB during early lactation [3,4]. Additionally, the incidences of metabolic diseases, such as ketosis, displaced abomasum, and reproductive disorders, such as retained placenta and susceptibility to infections, increase during early lactation in animals with severe NEB [5]. In this view, adipose tissue (AT) is a key regulator of metabolism in dairy cows [6]. Traditionally, AT was considered as a passive organ for the storage and mobilization of triglycerides during periods of excess or deficit of energy. However, for several years, AT and, more precisely, white AT is now considered as a dynamic endocrine tissue able to produce and secrete molecules called adipokines or adipocytokines [7,8]. These consist of polypeptides but also non-protein factors that are metabolically active molecules involved in different physiological functions, including immunity, metabolism, cardiovascular system regulation, and angiogenesis. In humans and rodents, adipokines regulate satiety, glucose and lipid metabolism, immune functions, angiogenesis, and reproductive functions [9]. In humans with the increased prevalence of obesity, these new roles of adipokines have spawned an increased interest in white adipose tissue. However, in dairy cows, little is known about the relation between adipose tissue proteins and the metabolic status in peripartum. Previous studies of adipose tissue of cows among parturition showed a differential abundance of proteins related to the insulin resistance phenomenon [10] and provided novel biomarkers for this condition. Using proteomic studies, Zachut et al. also identified novel biomarkers of heat stress in adipose tissue in late-pregnant cows [11]. However, proteomic studies on adipose tissue of dairy cows in relation to the net energy balance have not yet been performed. 

Our hypothesis took into consideration the possibility that profound metabolic changes are triggered in the cow adipose tissue at peripartum to overcome the NEB status. A massive energy amount is required to overcome the energy demand, and the mobilization of adipose tissue may play a key role. Individual variability in these mechanisms might be the cause of the different outcomes in terms of NEB status. In the present study, we provided a first proteomic survey of subcutaneous adipose tissue of dairy cows at peripartum. Proteomic profiles of cows with different scores of NEB (severe or low) at different stages of adipose tissue mobilization were comparatively evaluated. The findings of the current study contributed to the steadily increasing knowledge in the field of animal breeding management and opened new avenues in the research for the identification of novel biomarkers predictive of the animal energy status; thus, might imply the improvement of livestock breeding strategies by optimizing diet composition and management practice.

## 2. Materials and Methods

### 2.1. Animals and Experimental Design

All experimental protocols were approved by the ethics committee “Comité d’Ethique en Expérimentation Animale Val de Loire” (CEEA VdL, protocol registered as n° 2012-10-4) and were carried out in accordance with the guidelines of the French Council for Animal Care.

From a herd of 39 animals, we selected in the present study two sub-groups of animals either with low negative energy balance (lNEB, *n* = 6) or with severe negative energy balance (sNEB, *n* = 7) according to their energy balance (EB) at 1 week postpartum (EB < −13 Mcal/day for sNEB and EB > −2 Mcal/day for lNEB). Selected animals were reared in the same environment, and calving took place in the same period. The initial weight (one month before the calving) was 664 ± 15.5 for sNEB and 638 ± 17.9 kg for lNEB animals.

### 2.2. Body Weight, Milk Yield, Feeding, and Energy Balance

After each milking, cows were automatically weighted (software RIC version RW1.7, Hokofarm Group, Marknesse, The Netherland). Only the morning live body weight (LBW) was used for weight analyses because the afternoon body weight was more variable. All cows were milked twice daily. At the entrance of the milking parlor, the cows were identified by an electronic collar, and the milk yield of each cow was automatically recorded (software Manufeed 500 pro, vc5 version 2.011.14, Manus-Delaval, Elancourt, France). As LBW is affected by digestive contents, the estimation of empty body weight (EBW) was corrected for the digestive tract content. A change of 4.5 kg of digestive contents per kg of dry matter intake (DMI) was assumed [12]. Variation of EBW (VEBW) was calculated day after day: EBW of the previous day was taken as a reference weight [12]. Live body weight and VEBW as compared to one month before calving were measured from 4 weeks before calving until 16 weeks after calving in females. Dry matter intake was determined from the intake of fresh matter and the dry matter content of each feed of the ration. On average, there was one feeder for two cows. When a cow arrived in front of the feeder, it was recognized by a unique passive transponder attached to her ear tag. If the cow was allowed, the feeder opened, and the quantity of food eaten by the cow was automatically recorded (software RIC version RW1.7, Hokofarm Group, Marknesse, The Netherland). Dry matter intake was calculated daily from calving to week 16 after calving. Energy balance (expressed in Mcal/day) was calculated from calving to week 16 postpartum and corresponded to the difference between net energy intake and net energy needs for body maintenance and lactation.

### 2.3. Subcutaneous Adipose Tissue Thickness

Adipose tissue mobilization was assessed through subcutaneous fat thickness measurements in the sacral region using an ultrasonographic examination with a linear probe (LA 332 3.5/10.0-MHz transducer; Mylab30vet; Esaote, Hospimedi, Saint-Crépin-Ibouvillers, France). Backfat thickness was measured at one month and two weeks before calving, and at 1, 4, 8, 12, and 16 weeks postpartum. The variation of the backfat thickness relatively to week 4 before calving was calculated.

### 2.4. Biopsies of Adipose Tissue

Adipose tissue biopsies were carried out on the 6 animals with lNEB and the 7 animals with sNEB at one month before calving, one week after calving, and 16 weeks after calving. Cows had fasted for 12 h before surgery, and anesthesia was induced by intravenous (IV) injections of 12 to 14 mg of xylazine (Rompun^®^, Bayer, Leverkusen, Germany) and subcutaneous (SC) injections of 20 mg lidocaine (Lurocaïne^®^, Vetoquinol, Lure, France). Subcutaneous fat was collected from the dewlap under the neck, immediately frozen in liquid nitrogen, and stored at −80 °C until use. 

### 2.5. Plasma Non-Esterified Fatty Acids (NEFA) Assays

Blood samples were collected from the tail vein immediately before food distribution, once per week (from 4 weeks before calving until two months after calving) and twice a week (from 8 weeks until 16 weeks after calving). They were centrifuged at 3000× *g* for 10 min at 4 °C, and plasma was stored at −20 °C until its use for assays. Plasma NEFA was determined using enzymatic colorimetry assay (Wako Chemicals GmbH, Neuss, Germany). The intra- and inter-assay coefficient of variation of plasma NEFA measures were 6% and 7.8%, respectively. 

### 2.6. Proteomics

**Figure 1:**
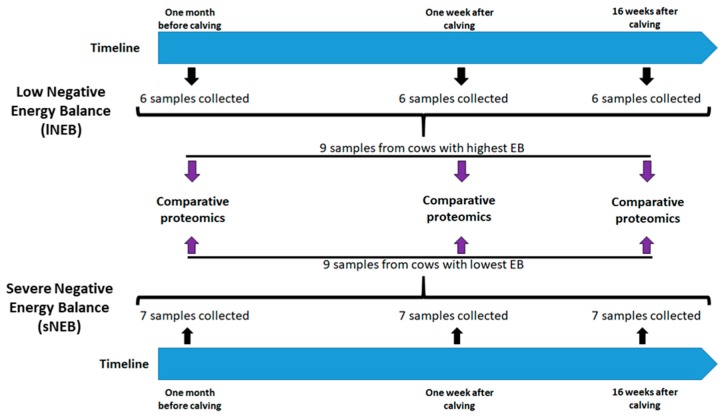
Experimental design and timeline of sample collection. The adipose tissues of two experimental groups (lNEB and sNEB) were analyzed in three different time points. Among the samples of each group, three were collected and analyzed for differential protein expression.

Proteomic profiles of adipose tissue samples derived from cows with a different score in NEB were analyzed. Adipose tissue from three animals with lNEB and three animals with sNEB was investigated in technical replicates over three time points for a total of 18 samples, nine belonging to the cows with the highest energetic balance, and nine belonging to the animals with the lowest energetic balance (Figure 1). The samples were collected from the same animal during the three time points that were, respectively, one month before calving, one week after calving, and 16 weeks after calving. Protein separation was performed using 2D SDS-PAGE, and differentially expressed proteins were identified by MALDI-TOF MS. 

### 2.7. Protein Samples Preparation for 2DE

The adipose tissue samples were dissolved in lysis buffer containing 7 M urea, 2 M thiourea, 2% (*w*/*v*) CHAPS, 2% Triton, 1× protease inhibitor cocktails, 1 mM Na_3_VO_4_, and 1 mM NaF. Thereafter, samples were processed by agitation with magnets for 2 h. Afterward, protein samples were centrifuged at 14,000 rpm for 30 min to remove insoluble materials. Thus, protein extracts were precipitated in Protein Bind tubes (Eppendorf) following a procedure adapted from Wesse and Fugge [13].

### 2.8. 2D SDS-PAGE 

Protein concentration in the samples was determined using Bradford assay, with BioRad protein assay stain and 2 µg/µL concentration of BSA as a standard. Optical density was measured using a spectrophotometer (Gene Quant 100, GE Healthcare, Chicago, Il, USA) at 595 nm. Proteins were separated using 2D SDS-PAGE. For isoelectric focusing (IEF) step, immobilized pH gradient (IPG) polyacrylamide gel strips (GE Healthcare, 7 cm, pH 3–10 NL) and Protean IEF Cell (Bio-Rad, Hercules, Ca, USA) were utilized. Prior to IEF, 100 µg of protein sample was dissolved in a solution containing 7 M urea, 2 M thiourea, 2% *w*/*v* CHAPS, 2% Triton, 30 mM DTT, 0.5% *w*/*v* ampholine (pH 3.5–10.0), and 1% *w*/*v* bromophenol blue. IPG strips were first actively rehydrated in the presence of the sample at 50 V and 20 °C for 17 h. After the rehydration step, paper wicks soaked in water were placed between cathode, anode, and gel strip for preventing high voltage to causing the burning of the strips. The voltage was gradually increased according to the following protocol: 100 V (1 h), 500 V (1 h), 1000 V (1 h), 2500 V (1 h), 5000 V until the cumulative voltage reached 50 kVh. A limitation of current up to 50 µA per gel strip was set. Following IEF, each strip was reduced for 15 min in 5 mL of solution containing 6 M urea, 2% *w*/*v* SDS, 50 mM Tris-HCl buffer, pH 8.8, and 20% *v*/*v* glycerol with 1% *w*/*v* DTT added, and then alkylated in 5 mL of the same solution with 2.5% *w*/*v* of iodoacetamide (IAA). IPG strips were then washed shortly in 1× running buffer (250 mM Tris-HCl, pH 8.8, 1920 mM glycine, 1% *w*/*v* SDS, and MilliQ water), loaded onto 12% *w*/*v* polyacrylamide resolving gels along with the protein ladder and fixed with 0.5% *w*/*v* agarose gel. The second dimension was carried out in a Mini-Protean Tetra system (Bio-Rad, Hercules, CA, USA). In the first step of electrophoresis, until the bromophenol blue front line entered the resolving gel, 8 mA per gel for 15 min was applied. In the second step, 16 mA per gel was applied until the bromophenol blue front line reached the bottom of the gel. Gels were then removed from the plates, washed three times for 5 min in 100 mL of deionized water, and left overnight to stain in 100 mL of preheated coomassie brilliant blue G-250 (Sigma–Aldrich, Milano, Italy).

Gel images were acquired using a flatbed scanner (ImageScanner III, GE Healthcare, Uppsala, Sweden) with a resolution of 600 dpi. Before scanning, gels were washed for 20 s in 70% *v*/*v* ethanol and then for 2 min in 100 mL of deionized water.

### 2.9. MALDI-TOF MS Analysis

According to Piras et al. [14,15], MALDI-TOF MS analysis was performed by Ultraflex III MALDI-TOF/TOF spectrometer (Bruker Daltonics, Macerata, Italy) in positive reflectron mode. Briefly, for the external calibration, the standard peptide mixture calibration was used (Bruker Daltonics, Macerata, Italy): *m*/*z*: 1046.5418, 1296.6848, 1347.7354, 1619.8223, 2093.0862, 2465.1983, 3147.4710), and to select monoisotopic peptide, MS spectra were analyzed by FlexAnalysis 3.3 software (Bruker Daltonics, Macerata, Italy).

After an internal calibration (known autolysis peaks of trypsin, *m*/*z*: 842.509 and 2211.104) and exclusion of contaminant ions (known matrix and human keratin peaks), the peak lists were analyzed by MASCOT v.2.4.1 algorithm (www.matrixscience.com) against SwissProt database released 2019_09 restricted to *Bos taurus* taxonomy (561,176 sequences).

For the peptide mass fingerprinting analysis (PMF), the query for database searching was set with these established parameters: carbamidomethylation of cysteines and oxidation on methionines as fixed modification and variable modification, respectively; one missed cleavage site for trypsin; maximal tolerance at 50 ppm. For protein identification assignment, only Mascot scores higher than 54 were considered significant (*p* < 0.05). 

To confirm PMF identifications, the instrument was switched in LIFT mode, and MS/MS spectra were acquired with 4–8 × 10^3^ laser shots using the instrument calibration file. For the fragmentation, precursor ions were manually selected, and the precursor mass window was automatically set. Using Flex-Analysis 3.3 software, each MS/MS spectra was processed by spectra baseline subtraction, smoothing (Savitsky–Golay), and centroiding. For database search analysis, the following parameters were used: carbamidomethylation of cysteines and oxidation on methionine among fixed and variable modifications, respectively; maximum of one missed cleavage; mass tolerance at 50 ppm for precursor ions and at a maximum at 0.4 Da for fragments. The database-dependent search was performed against the SwissProt database released 2019_09 (561,176 sequences) restricted to other Mammalia (13,206 sequences) and *Bos taurus* taxonomy. The confidence interval for protein identification was set to 95% (*p* < 0.05), and only peptides with an individual ion score above the identity threshold were considered correctly identified.

### 2.10. Statistical Analyses

Statistical analyses for dry matter intake, milk yield, live body weight, a variation of empty body weight, a variation of backfat thickness, energy balance, and plasma NEFA were performed with SAS^®^ software version 9.4. Unless differently specified, data were analyzed using the MIXED procedure for linear mixed models. A repeated effect of time (the week before and after calving) within animals was tested. The residuals from the observations generated from the mixed models were tested for normal distribution. 

The model used was:

Y*_ijk_* = µ + NEB*_i_* + Week*_j_* + NEB*_i_* × Week*_j_* + e*_ijk_*

where Y*_ijkl_* is the dependent variable (dry matter intake, milk yield, live body weight, variation of empty body weight, variation of backfat thickness, energy balance, plasma), µ is the overall mean, NEB*_i_* is the fixed effect of NEB *i* (*i* = lNEB, hNEB), Week*_j_* is the fixed effect of week *j* (*j* = 17 classes, 19 classes), NEB*_i_* × Week*_j_* is the interaction between NEB*_i_* and Week*_j_*, and e*_ijk_* is the residual error. 

Least square means (LSmeans, ± standard error of the mean, SEM) estimated by the models were adjusted using the Scheffe adjustment for multiple-post ANOVA comparisons and compared. 

Differences with corresponding *p*-values, *p* < 0.05, were considered as significant.

Variations in protein expression between lNEB cows and sNEB cows were analyzed using the Progenesis SameSpots software (Nonlinear Dynamics, Newcastle upon Tyne, UK), Version 4.6. After evaluating the quality of the images, the module for 2D gel analysis was used to align the images, subtract background, detect, normalize, and match spots. All spots were then manually reviewed and selected for excision. 

Statistical analysis was performed using the Progenesis Stats module on the log-normalized volumes for all spots. Stats module performed automatically a one-way ANOVA on each spot to evaluate the *p*-value between different groups, and the *p*-values under 0.05 were considered statistically significant. 

### 2.11. Bioinformatics Analysis

The functional classification of the identified proteins was performed using the PANTHER classification system [16]. The Gene Ontology (GO) slim biological processes related to the overrepresented and underrepresented proteins were obtained through the gene list analysis module.

High confidence (score 0.700) protein-protein interactions and relations between proteins were investigated via the STRING database of protein-protein interactions [17] using the accession numbers of all differentially expressed proteins. 

## 3. Results

**Figure 2:**
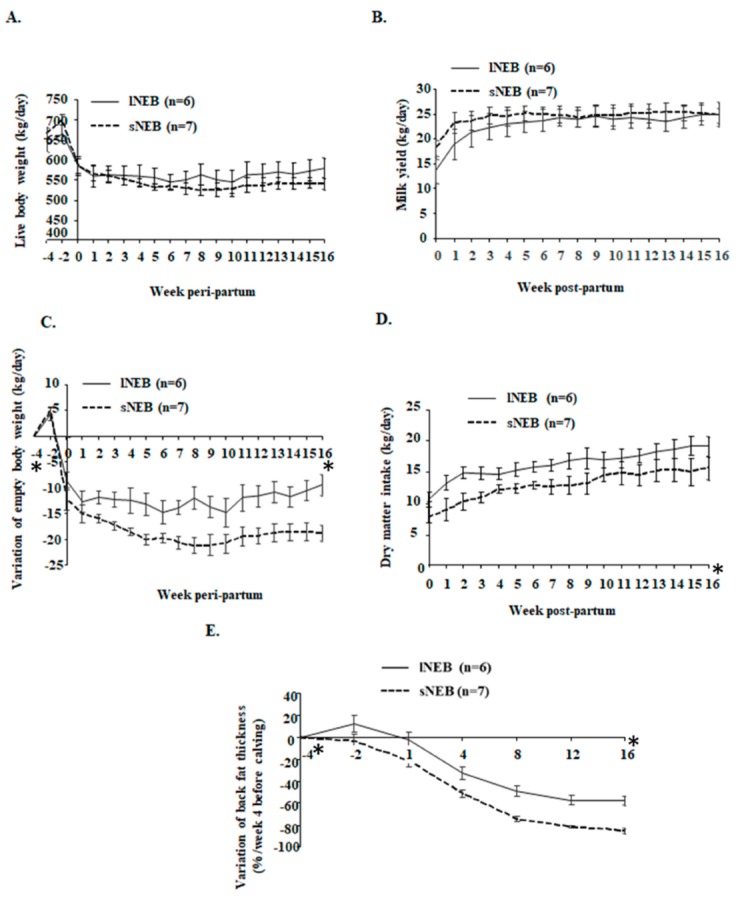
Live body weight (**A**), milk yield (**B**), variation of empty body weight (**C**), dry matter intake (**D**), and variation of backfat thickness (**E**) in Holstein cows with lNEB (*n* = 6) and sNEB (*n* = 7) at week 4 before calving and week 16 after calving. Results are presented as LSmeans ± SEM. Asterisks (*) are representative of statistically significant (*p* < 0.05) differences among LSmeans of the two groups. LSmeans, least square means; SEM, standard error mean.

**Figure 3:**
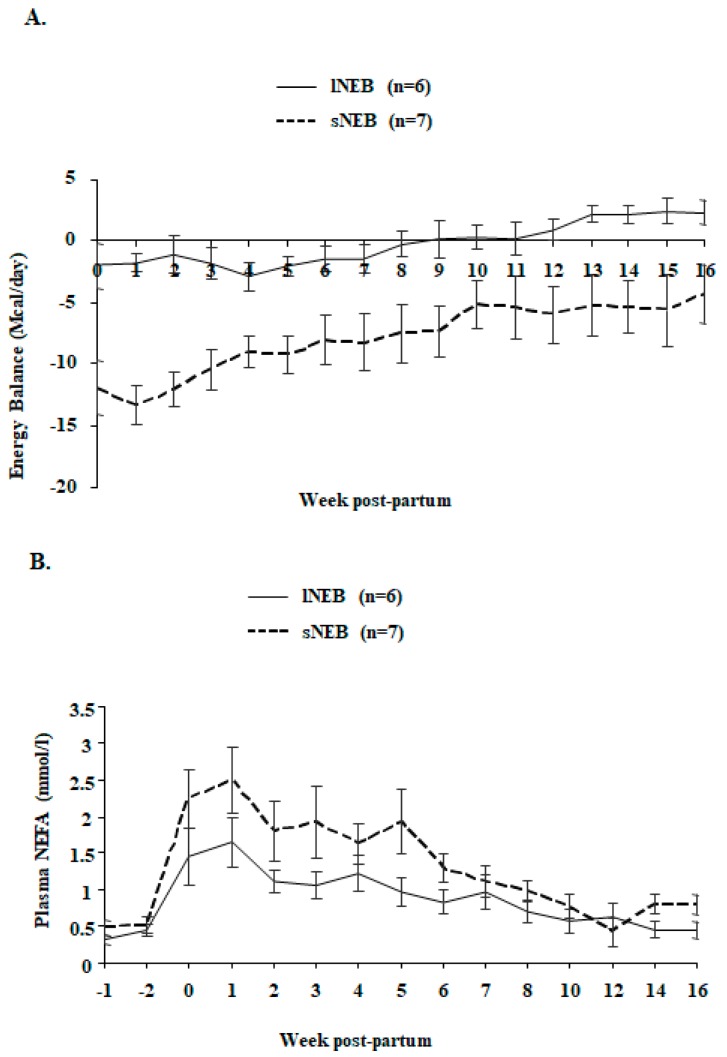
Energy balance (**A**) and plasma NEFA (non-esterified fatty acids) (mmol/l) (**B**) in Holstein cows with lNEB (*n* = 6) or sNEB (*n* = 7) at week 4 before calving and week 16 after calving. Results are presented as LSmeans ± SEM.

Adipose tissue samples were collected from cows in lNEB and cows in sNEB. The classification of samples based on energy balance was available only at 1 and 16 weeks postpartum when the EB level could be measured according to milk yield (Figure 2 and Figure 3). Therefore, samples of one month before calving were collected as well and stored at −80 °C until the proper classification was known. Three samples of each group (three with the highest positive lNEB and three with the lowest negative sNEB) at each time point were chosen for the comparative proteomics analysis, as described in Figure 1.

Live body weight, a variation of empty body weight, dry matter intake, milk yield, a variation of backfat thickness, energy balance, and plasma NEFA are depicted in Figure 2, Figure 3, and resumed in Table 1. As shown in Figure 2 and Table 1, live body weight (Figure 2A) and milk yield (Figure 2B) were changing during the time points (*p* < 0.0001), but there were no differences among EB groups from calving to week 16 postpartum. However, significant differences among least-square means of sNEB and lNEB groups were found in the samples collected at week 4 before calving and the samples collected at week 16 after calving. They consisted of the variation of empty body weight (for NEB effect, difference hNEB-lNEB: −6.47 kg/day, *p* = 0.041, Figure 2C), dry matter intake (for NEB effect, difference hNEB-lNEB: −3.36 kg/day, *p* = 0.002, Figure 2D), backfat thickness (for NEB effect, difference hNEB-lNEB: −18.51%, *p* < 0.001, Figure 2E), and plasma NEFA (for NEB effect, difference hNEB-lNEB: 0.42 mmol/l, *p* = 0.042, Figure 3B). 

**Figure 4:**
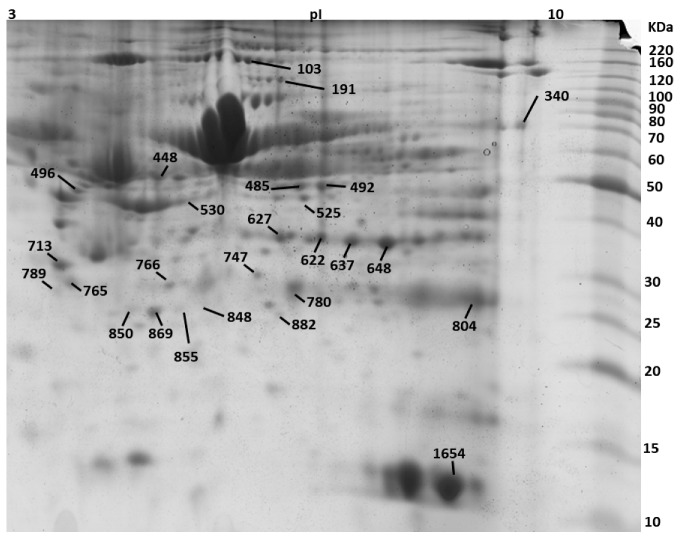
Selected gel spots from a representative 2DE gel.

**Figure 5:**
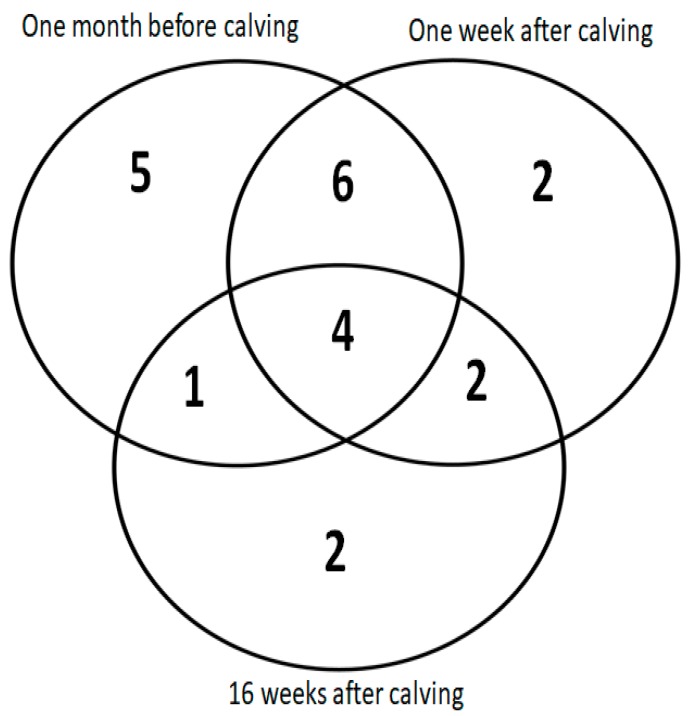
Venn diagram representing the distribution of differentially expressed proteins. This Venn diagram represents the differential expression of the data represented in Table 3 of the differential protein expression of the sNEB group vs. lNEB group.

Two-dimensional electrophoresis experiments were performed on biological triplicates for each experimental group (Figure 1) and allowed the detection and measurement of around 700 proteoforms. Spot selection based on a changing 2DE pattern among sample groups resulted in 26 statistically significant proteins (Figure 4) and the final identification of 22 proteins. A representation of the selected gel spots along with a table summarizing the obtained MS data is provided as Figure 4 and Table 2, respectively. Identified proteins were also sorted in a Venn diagram, representing all the proteins that were differentially expressed and identified between the peripartum periods and between groups of lNEB and sNEB (one-way ANOVA, *p*-value < 0.05, Figure 5). All differentially expressed proteins commonly shared among comparisons followed the same trend (Table 3), confirming their effective role in the NEB response. 

Among these, four key proteins, common to all investigated time points, were shown to be differentially expressed, regardless of the time points, between both lNEB and sNEB groups, namely annexin, actin-related protein 10, glyceraldehyde-3-phosphate dehydrogenase, and fatty acid-binding protein. Two different isoforms of annexin A1 were underrepresented one month before calving with a fold change of at least two. The underexpression of one of these isoforms was maintained one week after calving, and it returned to a steady state 16 weeks after calving. Differential expression of annexin A2 was the same among all comparisons with a consistent decreasing fold change between one month before calving and the other two time points.

The comparison between lNEB and sNEB one month before calving highlighted 16 proteins differentially expressed; among them, two were overrepresented and 14 underrepresented.

The comparison between lNEB and sNEB of the samples collected one week after calving highlighted a total of 14 proteins with a different abundance profile, among which five overrepresented and nine underrepresented. The same comparison revealed nine differentially expressed proteins between lNEB and sNEB 16 weeks after calving. Among these, four were overrepresented, and five were underrepresented. 

**Figure 6:**
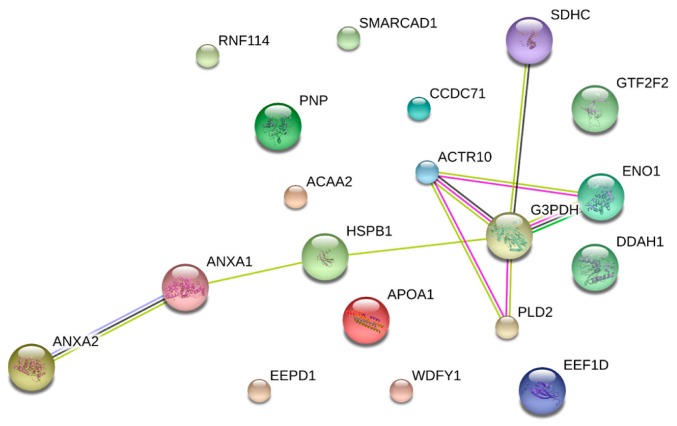
String protein-protein interaction analysis. The figure highlights the connections between differentially represented proteins.

To increase the depth of analysis and to be able to illustrate better the biology of the rearrangement of adipose tissue in the peripartum, we also analyzed all class and all proteins through PANTHER GO slim analysis. Moreover, their possible interaction was analyzed through the STRING protein-protein interaction network (Figure 6).

The interaction analysis showed the link between eight of the differentially expressed proteins, and seven out of eight were less abundant in this biological system. Moreover, all the proteins connected by STRING were differentially expressed in at least two of the three time points, except for the succinate dehydrogenase cytochrome b560 subunit mitochondrial that was highly abundant just one week after calving.

**Figure 7:**
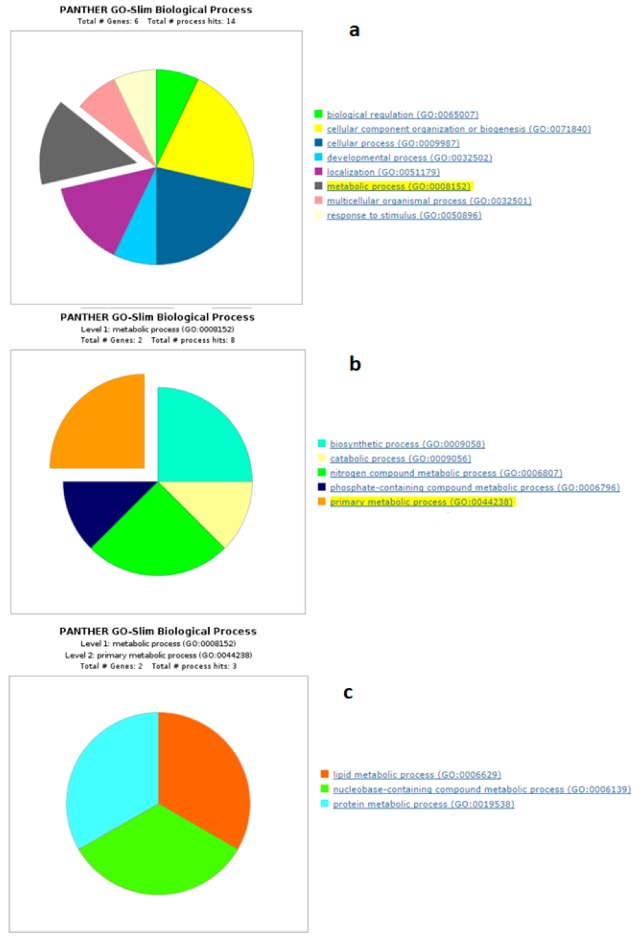
Functional classification of overrepresented proteins among all time points between sNEB and lNEB with the enrichment of most represented processes up to the primary metabolic process. (**a**) Biological process, (**b**) metabolic process, (**c**) primary metabolic process.

**Figure 8:**
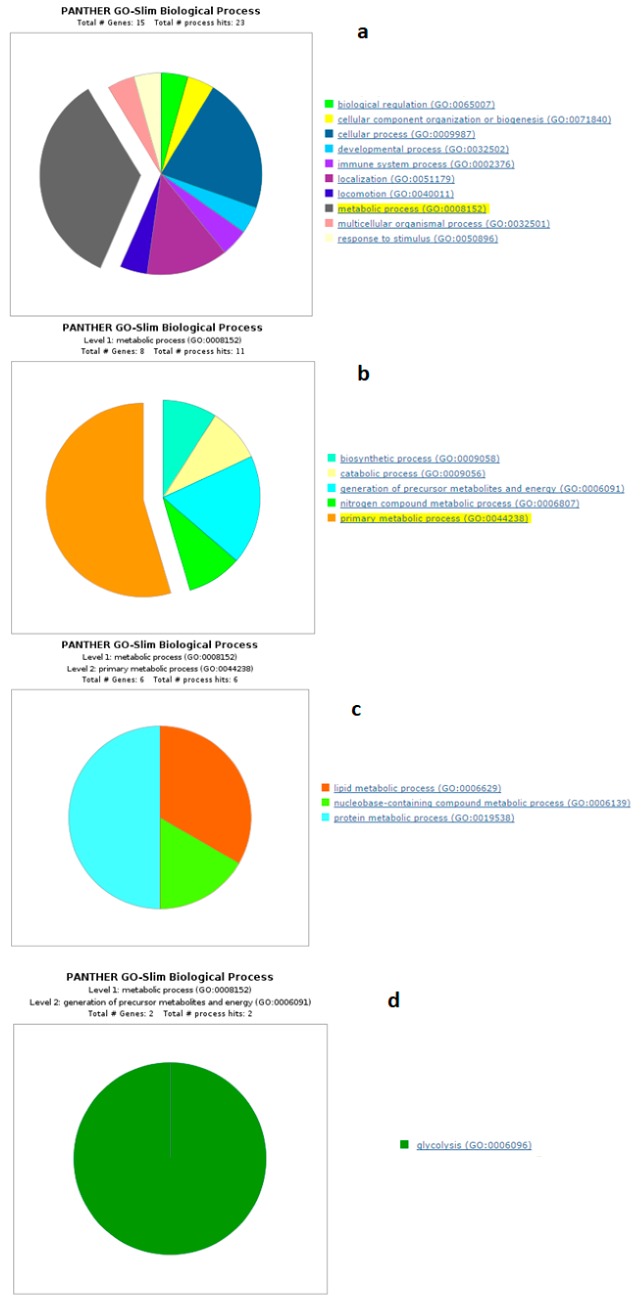
Functional classification of underrepresented proteins of all time points between sNEB and lNEB with the enrichment of most represented processes up to the primary metabolic process. (**a**) Biological process, (**b**) metabolic process, (**c**) primary metabolic process (**d**) generation of precursor metabolites and energy.

Panther GO analysis highlighted the most representative biological processes in which the differentially expressed proteins were involved. The number of underrepresented proteins was higher than the overrepresented ones. The GO analysis highlighted that overrepresented proteins in sNEB were involved in a greater variety of metabolic processes, including primary metabolic processes (Figure 7 and Figure 8).

## 4. Discussion

Our data suggested that there was a consistent difference in the metabolic profile among lNEB and sNEB experimental groups. By comparing the differentially expressed proteins between both groups, four proteins were common in all time points and constant in their differential abundance during the peripartum, suggesting annexin A2, actin-related protein 10, glyceraldehyde-3-phosphate dehydrogenase, and fatty acid-binding (FABP) protein, together with rates of fat tissue mobilization, as important players for the different response to negative energy balance among experimental groups. Interestingly, the FABP family is the master switch of the PPAR signaling pathway [18], and its constant overrepresentation in sNEB during all sampled periods indicated a pivotal role of the PPAR signaling pathway in the metabolic changes occurring in the sNEB phase.

### 4.1. Biological Processes of Highly Abundant Proteins

Among all differentially expressed proteins, six were found to be overrepresented in the comparisons over all the time points. One of them (fatty acid-binding protein) was highly abundant in sNEB in the time point lapse ranging from one month before calving up to 16 weeks after calving. Apolipoprotein A-I was strongly overrepresented in the period after calving (one week to 16 weeks). It is the major component of high-density lipoprotein(HDL), and its major role is related to the transport of fat molecules from the cells. Its overrepresentation in sNEB cows after calving follows the trend of FABP4, suggesting both proteins as active players in resuming the impaired function of adipose tissue of sNEB cows. According to previously published experimental evidence on a mouse model [19], ApoA-I overexpression seems to be positively linked to energy expenditure, the faster reduction of white fat mass, and improved insulin sensitivity. Around calving, in order to compensate for the lack of energy, fat is mobilized from the adipose tissue in the form of non-esterified fatty acids to be transported to different organs. Accordingly, a previous study of Folnožić and colleagues on Holstein-Friesian dairy cows reported an increased lipid mobilization after calving [20]. 

The non-esterified fatty acid can be again converted into triacylglycerol (TAG) from the liver and stored in hepatocytes. This is mainly the reason why up to 40% of dairy cows develop fatty liver disease and could explain as well such an increased amount of ApoA-1 in adipose tissue. Based on the evidences described by Karavia and colleagues [21] on a mouse model and on the assumption that ApoA-1 is one of the major protein components of HDL, it is consistent to assume that its overexpression is due to the higher amount of NEFA in blood, exactly like in these animals, as well as to a required faster clearance of fatty acids from the liver. In this view, Turk et al. already reported a markedly affected concentration of triglycerides, total cholesterol, HDL, beta-hydroxybutyrate, free fatty acids, and paraoxonase-1 activity in heifers throughout transition period [22,23]. 

Fatty acid-binding protein’s main function is related to lipid transport and has the capability to bind with high-affinity hydrophobic ligands as saturated and unsaturated long-chain fatty acids and eicosanoids (leukotrienes and prostaglandins) [24]. Its strong overrepresentation during all the time points around parturition period could reflect the overall condition of lipid mobilization and faster lipid metabolism, in the attempt to provide energy that attenuates the sNEB.

Fatty acid-binding protein 4 (FABP4) is mainly expressed in adipocytes and macrophages. It is responsible for the development of insulin resistance and inflammation [25], it can reversibly bind to hydrophobic ligands, such as saturated and unsaturated long-chain fatty acids (FAs), and transport FAs to specific organelles in the cell [26]. Normally, the amount of FABP in cells is proportional to the rates of FA metabolism [27]. Its overexpression in our model is consistent and correlates with NEB, and it is indeed overexpressed in the sNEB group. A higher metabolism of adipose tissue is also in agreement with the theory of Vries and Veerkamp that describes how a decrease in fat deposit percentage during early lactation might serve as an indicator of energy balance [28].

### 4.2. Biological Processes of Less Abundant Proteins

Most of the underrepresented proteins were assigned to a variety of metabolic processes. Expanding the section of the lipid metabolic process, it was possible to observe that the underrepresented proteins involved in this biological process were annexin A1 and annexin A2. Relevant experimental evidence documents the key role of these proteins in the regulation of fat tissue metabolism. One of its major roles is linked to fat accumulation in cows [29]. We found a positive correlation between the annexin A1 levels and the backfat accumulation. Likewise, annexin A2 deficiency has been linked to white adipose tissue hypotrophy due to reduced fatty acid uptake by endothelium and adipocytes [30]. In our experimental design, annexins A1 and A2 were both strongly underrepresented in severe negative energy balance cows. Considering the previously cited experimental evidence, it seems that annexins A1 and A2 are positively involved in fat storage and adipose tissue formation. Their reduced abundance in sNEB cows might be due to the reduced capability to fulfill the energy requirements necessary during pregnancy and lactation and undertake a fast mobilization of adipose reserves that are not supported by the expression of annexins.

Insulin resistance could represent another important feature to take into account. The differential protein expression of adipose tissue in cows has been evaluated in relation to insulin resistance [10]. We evaluated the differential protein expression of adipose tissue from cows grouped according to insulin resistance or sensitivity following the evaluation of protein kinase B phosphorylation after insulin stimulation. The outcome highlighted that annexin A1 was highly abundant in insulin-resistant adipose tissue and, other experimental shreds of evidence, highlighted as well, its positive relation with backfat thicknesses [29]. These two experimental pieces of evidence could explain why sNEB adipose tissue is faster in lipid metabolism and less efficient in energy storage.

The generation of precursor metabolites and energy highlighted in the panther pie chart (Figure 8b) were composed of two main proteins, and both were underrepresented and part of glycolysis metabolism (Figure 8d). The first one was alpha-enolase, whose overexpression was particularly lower one month before calving, and then the difference decreased up to become non-significant 16 weeks after calving. The second one was the glyceraldehyde-3-phosphate dehydrogenase, whose reduced abundance was accentuated in sNEB in all the time points, but less relevant in the period close or after parturition. The reduced abundance of these two proteins seemed to demonstrate the decreasing amount of the glycolytic process. It was in agreement with the decreased expression of annexin 2, which is also involved in the translocation of GLUT 4 to the membrane for glucose intake [31]. All this evidence highlighted a strong decrease in glucose metabolism in the adipose tissue of sNEB.

Actin-related protein 10 was another protein that was found to be less abundant among all the time points in sNEB. Its role is mainly related to intracellular trafficking and microtubule-based movement. It was again in agreement with the previously mentioned theory about an sNEB adipose tissue more oriented to lipid transport in other organs and then to energy storage.

## 5. Conclusions

**Figure 9:**
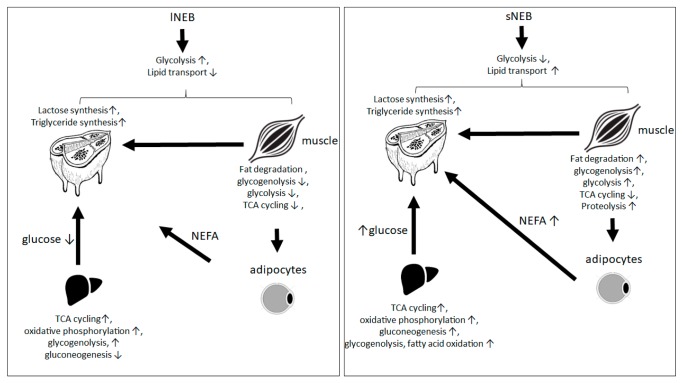
Resuming scheme of different NEB levels in the physiology of lactating cows. The represented arrows represent the trend of the de-regulated metabolisms, pathways, and metabolites in sNEB cows in comparison to lNEB cows. Up arrow (↑) and down arrow (↓) refer to increased and decreased concentrations/activity, respectively.

The results obtained showed several differences in the protein profile involved in biological processes and pathways, as glycolysis and lipid metabolism and transport. The most important differences highlighted that glycolysis was dramatically underrepresented in adipocytes of sNEB cows, and, on the contrary, lipid transport out of adipocytes was highly increased (Figure 3B). This observation could be explained by the impairment in lipid biosynthesis and in the fatty acid mobilization that was consistent with the level of NEB. The hypothesized mechanism behind this phenomenon was resumed in Figure 9, representing the combination of obtained results and previous experimental evidence, according to Kuhla and Metges [32]. 

The major requirement of energy is due to the necessity of lipids necessary for milk production. The adipose tissue of sNEB cows seems to be more efficient in lipid transport and mobilization and less efficient in the glycolytic pathway. This may produce a faster metabolism of adipose tissue of sNEB cows that are as well faster in depleting the fat deposit [33]. This different efficiency in adipose tissue metabolism might be relevant for the complications due to the NEB among calving and play a key role in the timing necessary for recovering. 

## Figures and Tables

**Table 1:** Management and nutritional parameters for the two groups of animals (severe Negative Energy Balance (*n* = 7), and low Negative Energy Balance (*n* = 6)). Results are presented as LSmeans. *p*-values of the effects of NEB, week peripartum, interaction between Negative Energy Balance, and week peripartum.

Group	lNEB		sNEB		*p*-Values		
	Lsmeans	SEM	Lsmeans	SEM	NEB	Week	NEB × Week
Live body weight, kg/day	560.56	0.66	540.26	0.78	0.649	<0.0001	0.822
Variation of empty body weight, kg/day	−12.19	0.10	−18.66	0.11	0.041	<0.0001	0.774
Milk yield, kg/day	22.92	0.11	24.29	0.07	0.364	<0.0001	0.004
Dry matter intake, kg/day	16.10	0.07	12.74	0.12	0.002	<0.0001	0.004
Energy balance, Mcal/day	−0.26	0.08	−7.92	0.11	<0.0001	<0.0001	0.189
Variation of backfat thickness (%/week 4 before calving)	−26.75	1.04	−45.26	0.85	<0.001	<0.0001	0.494
Non Esterified Fatty Acids, mmol/L	0.86	0.02	1.28	0.04	0.042	<0.0001	0.485

**Table 2:** Summary of the MS-data for protein identification. ^a^ Name of identified proteins; ^b^ Accession No. according to Swiss-Prot database; ^c^ Peptide mass fingerprinting (PMF) score calculated by MASCOT 2.4.1 algorithm (www.matrixscience.com) after database search; ^d^ Sequence coverage; ^e^ Number of experimental peptides matched versus searched peptides; ^f^ Aminoacidic sequence of the peptides identified by MS/MS analysis, and ^g^ Related score calculated by MASCOT 2.4.1 algorithm. The underlined letter indicates oxidation of methionine residue; ^h^ Monoisotopic masses of the parent ions used for MS/MS analysis.

Spot No.	Protein Description ^a^	Accession No. ^b^	Theoretical Mr(kDa)/pI	PMF			Tandem Mass Spectrometry		
				Mascot Score ^c^	% Seq. Coverage ^d^	Matched Peptides ^e^	Peptide Sequence ^f^	Mascot Score ^g^	*m*/*z*^h^
103	SWI/SNF-related matrix-associated actin-dependent regulator of chromatin subfamily A containing DEAD/H box 1 OS = Bos taurus	E1B7X9	118/5.5	74	14	Nov-81	^801-^EMSQLMLK^-808^	86	78
							^763-^SINNMEKNTEMCNVMMQLR^-781^	979.49	2302.007
191	Phospholipase D2 OS = Bos taurus GN = PLD2 PE = 2 SV = 1	Q0V8L6	106.6/7.8	72	12	14/41	^558-^HFIQRWNFTK^-567^	76	1376.73
							^419-^ALMLLHPNIKVMR^-431^	82	1551.96
340	3-ketoacyl-CoA thiolase, mitochondrial OS = Bos taurus	Q3T0R7	42.6/8.06	64	29	26-Oct	^333-^SLNLDPSK^-340^	96	873.43
							^77-^VGIPKETPAITINR^-90^	98	1508.8
448	Endonuclease/exonuclease/phosphatase family domain-containing protein 1 OS = Bos taurus	Q3MHJ7	63.2/8.6	71	24	Oct-61	^154-^GLTEKMAVSIVDYR^-167^	102	1597.82
							^40-^LNINTATEEELMTLPGVTR^-58^	86	2118.81
485	WD repeat and FYVE domain-containing protein 1 OS = Bos taurus GN = WDFY1 PE = 2 SV = 1	Q2KIY3	47.29/6.9	76	35	24-Sep	^109-^TYPAHQNR^-116^	92	986.5
							^104-^MNFIKTYPAHQNR^-116^	106	1619.7
492	Alpha-enolase OS = Bos taurus GN = ENO1 PE = 1 SV = 4	Q9XSJ4	47.64/6.37	132	35	13/39	^240-^VVIGMDVAASEFYR^-253^	126	1556.8
							^163-^LAMQEFMILPVGAENFR^-179^	104	1965.98
530	Actin, cytoplasmic 1 OS = Bos taurus GN = ACTB PE = 1 SV = 1	P60712	42.05/5.29	94	28	Oct-47	^197-^GYSFTTTAER^-206^	86	1132.96
							^360-^QEYDESGPSIVHR^-372^	74	1516.66
622	Annexin A1 OS = Bos taurus GN = ANXA1 PE = 2 SV = 2	P46193	63.2/8.6	98	46	15/74	^154-^GLTEKMAVSIVDYR^-167^	102	1597.82
							^40-^LNINTATEEELMTLPGVTR^-58^	86	2118.81
627	Annexin A1 OS = Bos taurus GN = ANXA1 PE = 2 SV = 2	P46193	63.2/8.6	66	30	13/74	^119-^DAEELRAAMK^-129^	76	1071,55
							^154-^GLTEKMAVSIVDYR^-167^	78	1597.82
637	Annexin A2 OS = Bos taurus GN = ANXA2 PE = 1 SV = 2	P04272	38.8/6.2	106	49	Nov-81	^48-^TKGVDEVTIVNILTNR^-63^	122	1771.86
							^314-^SLYYYIQQDTKGDYQK^-329^	102	2012.91
648	Actin-related protein 10 OS = Bos taurus GN = ACTR10 PE = 2 SV = 1	Q3ZBD2	46.84/7.06	86			^404-^NQPPLMKR^-411^	82	999.61
					36	26/109	^279-^SVATLILDSLMQCPIDTR^-296^	70	2012.91
713	Coiled-coil domain-containing protein 71 OS = Bos taurus GN = CCDC71 PE = 2 SV = 1	Q2HJ91	48.08/5.13	76	31	Nov-87	^220-^LGNAQLKAPR^-229^	78	1067.68
							^198-^AQSLQLSLGDSPLKVR^-213^	96	1712.08
747	Purine nucleoside phosphorylase OS = Bos taurus GN = PNP PE = 1 SV = 3	P55859	32.24/5.92	90	28	20-Aug	^68-^LVFGILNGR^-76^	92	988.59
							^40-^DHINLPGFSGENPLR^-58^	86	2118.81
765	Elongation factor 1-delta OS = Bos taurus	A5D989	30.97/5.42	82	36	Jul-57	^1-^MATNFLVHEK^-10^	82	1189.691
							^259-^SHQVEEHVQSVDIAAFNKI^-277^	80	2151.12
766	Glyceraldehyde-3-phosphate dehydrogenase OS = Bos taurus GN = GAPDH PE = 1 SV = 4	P10096	36.07/8.5	66	32	May-57	^131-^MGVNHEKYN-140	66	929.27
							^171-^GLMTTVHAIT ATQKTVDGPS^-190^	88	1615.88
780	E3 ubiquitin-protein ligase RNF114 OS = Bos taurus	Q4U5R4	26.65/6.5	96	36	Oct-64	^141-^YTFPCPYCPEK^-151^	76	1463.61
							^11-^DGGAQLAGPAAEADPLGR^-28^	98	1665.74
789	N(G),N(G)-dimethylarginine dimethylaminohydrolase 1 OS = Bos taurus GN = DDAH1 PE = 1 SV = 3	P56965	31.6/5.7	84	58	12-Jul	^150-^GAEILADTFK^-159^	78	1063.55
							^231-^GHVLLHRTPEEYPESAK^-247^	84	1963.08
848	Succinate dehydrogenase cytochrome b560 subunit, mitochondrial OS = Bos taurus GN = SDHC PE = 1 SV = 2	P35720	18.7/9.8	76	44	14-Jun	^8-^HVGRHCLR^-15^	67	1034.52
							^18-^NLGSNRPLSPHITIYR^-36^	97	1836.99
850	General transcription factor IIF subunit 2 OS = Bos taurus GN = GTF2F2 PE = 2 SV = 1	Q2T9L9	28.49/9.24	83	29	30-Aug	^26-^YLSQQWAK^-33^	86	1023.57
							^208-^QPVSYLKDILK^-218^	92	1303.8
855	Fatty acid-binding protein, adipocyte OS = Bos taurus GN = FABP4 PE = 1 SV = 4	P48035	15.35/7.57	96	39	Dec-60	^11-^WRLVESK^-17^	76	917.5
							^111-^LEDGKLVVVCVMNNVTCTR^-129^	98	2225.11
869	Apolipoprotein A-I OS = Bos taurus GN = APOA1 PE = 1 SV = 3	P15497	30.25/5.71	118	39	14/54	^142-^VAPLGEEFR^-150^	102	1017.53
							^131-^WHEEVEIYR^-159^	98	1260.57
882	Heat shock protein beta-1 OS = Bos taurus GN = HSPB1 PE = 2 SV = 1	Q3T149	22.43/5.98	90	36	23-Jul	^29-^LFDQAFGLPR^-38^	96	1163.97
							^13-^GPSWDPFRDWYPAHSR^-28^	92	1973.91

OS: Organism; GN: gene name; PE: Protein existence; SV: Sequence version.

**Table 3:** Differentially expressed proteins in severe negative energy balance and low negative energy balance.

Uniprot Name/Accession Number	String Name	Same Spots Coding Number	Protein Name	One Month before Calving: Trend/Fold Change/*p*-Value	One Week after Calving: Trend/Fold Change/*p*-Value	16 Weeks after Calving: Trend/Fold Change/*p*-Value
E1B7X9	SMARCAD1	103	SWI/SNF-related matrix-associated actin-dependent regulator of chromatin subfamily A containing DEAD/H box 1 OS = Bos taurus	↓/1.9/0.0193		
Q0V8L6	PLD2	191	Phospholipase D2 OS = Bos taurus GN = PLD2 PE = 2 SV = 1	↓/2.1/0.004		↓/2.3/0.030
Q3T0R7	ACAA2	340	3-ketoacyl-CoA thiolase, mitochondrial OS = Bos taurus			↓/3.3/0.028
Q3MHJ7	EEPD1	448	Endonuclease/exonuclease/phosphatase family domain-containing protein 1 OS = Bos taurus	↑/1.6/0.0086	↑/1.5/0.0072	
Q2KIY3	WDFY1	485	WD repeat and FYVE domain-containing protein 1 OS = Bos taurus GN = WDFY1 PE = 2 SV = 1		↓/1.6/0.0207	
Q9XSJ4	ENO1	492	Alpha-enolase OS = Bos taurus GN = ENO1 PE = 1 SV = 4	↓/1.8/0.0004	↓/1.4/0.050	
P60712	NA	530	Actin, cytoplasmic 1 OS = Bos taurus GN = ACTB PE = 1 SV = 1		↑/1.7/0.0297	↑/2/0.0080
P46193	ANXA1	622	Annexin A1 OS = Bos taurus GN = ANXA1 PE = 2 SV = 2	↓/2/0.00186		
P46193	ANXA1	627	Annexin A1 OS = Bos taurus GN = ANXA1 PE = 2 SV = 2	↓/2.9/0.0429	↓/1.8/0.0502	
P04272	ANXA2	637	Annexin A2 OS = Bos taurus GN = ANXA2 PE = 1 SV = 2	↓/2.7/0.0118	↓/1.7/0.0085	↓/1.8/0.0098
Q3ZBD2	ACTR10	648	Actin-related protein 10 OS = Bos taurus GN = ACTR10 PE = 2 SV = 1	↓/2.1/0.0050	↓/1.7/0.0085	↓/1.4/0.038
**Uniprot Name/Accession Number**	**String Name**	**SameSpots Coding Number**	**Protein Name**	**One Month before Calving: Trend/Fold Change/*p*-Value**	**One Week after Calving: Trend/Fold Change/*p*-Value**	**16 Weeks after Calving: Trend/Fold Change/*p*-Value**
Q2HJ91	CCDC71	713	Coiled-coil domain-containing protein 71 OS = Bos taurus GN = CCDC71 PE = 2 SV = 1	↓/1.2/0.0026		
P55859	PNP	747	Purine nucleoside phosphorylase OS = Bos taurus GN = PNP PE = 1 SV = 3	↓/1.4/0.0439	↓/1.3/0.0165	
A5D989	EEF1D	765	Elongation factor 1-delta	↓/1.6/0.00141		
P10096	G3PDH	766	Glyceraldehyde-3-phosphate dehydrogenase OS = Bos taurus GN = GAPDH PE = 1 SV = 4	↓/1.8/0.0201	↓/1.6/0.0260	↓/1.2/0.040
Q4U5R4	RNF114	780	E3 ubiquitin-protein ligase RNF114 OS = Bos taurus	↓/1.3/0.024		
P56965	DDAH1	789	N(G),N(G)-dimethylarginine dimethylaminohydrolase 1 OS = Bos taurus GN = DDAH1 PE = 1 SV = 3	↓/2.2/	↓/1.8/0.0011	
				8.84 × 10^−5^		
P35720	SDHC	848	Succinate dehydrogenase cytochrome b560 subunit, mitochondrial OS = Bos taurus GN = SDHC PE = 1 SV = 2		↑/1.8/0.0059	
Q2T9L9	GTF2F2	850	General transcription factor IIF subunit 2 OS = Bos taurus GN = GTF2F2 PE = 2 SV = 1			↑/3/0.0104
P48035	NA	855	Fatty acid-binding protein, adipocyte OS = Bos taurus GN = FABP4 PE = 1 SV = 4	↑/2.2/0.0053	↑/2.7/0.0231	↑/3.3/0.0127
P15497	APOA1	869	Apolipoprotein A-I OS = Bos taurus GN = APOA1 PE = 1 SV = 3		↑/3/0.0028	↑/2.6/0.0343
Q3T149	HSPB1	882	Heat shock protein beta-1 OS = Bos taurus GN = HSPB1 PE = 2 SV = 1	↓/2.8/0.0122	↓/2.7/0.005	

Table of differentially expressed proteins. ↑: proteins overrepresented in high negative energy balance. ↓: proteins underrepresented in high negative energy balance. OS: Organism; GN: gene name; PE: Protein existence; SV: Sequence version.
